# Cardiovascular disease risk assessment in patients with familial Mediterranean fever related renal amyloidosis

**DOI:** 10.1038/s41598-020-75433-7

**Published:** 2020-10-27

**Authors:** Micol Romano, David Piskin, Roberta A. Berard, Bradley C. Jackson, Cengizhan Acikel, Juan J. Carrero, Helen J. Lachmann, Mahmut I. Yilmaz, Erkan Demirkaya

**Affiliations:** 1 Division of Paediatric Rheumatology, Department of Paediatrics, Schulich School of Medicine & Dentistry, University of Western Ontario, 800 Commissioners Rd E. B1-146, London, ON N6A 5W9 Canada; 2 Department of Pediatric Rheumatology, ASST G Pini, Milano, Italy; 3 Lawson Health Research Institute, London Health Sciences Center, London, ON Canada; 4 CRI - Clinical Research International Ltd., Cologne, Germany; 5 Department of Medical Epidemiology and Biostatistics (MEB), Karolinska Institutet, Stockholm, Sweden; 6 Division of Medicine (Royal Free Campus), Centre for Amyloidosis & Acute Phase Proteins, London, UK; 7 Unit of Nephrology, Epigenetic Health Solutions, Ankara, Turkey; 8 Department of Epidemiology and Biostatistics, Schulich School of Medicine & Dentistry, University of Western Ontario, London, ON Canada

**Keywords:** Chronic kidney disease, Cardiovascular diseases, Rheumatology

## Abstract

Chronic inflammation and proteinuria is a risk factor for cardiovascular disease (CVD) in patients with chronic kidney diseases and rheumatologic disorders. Our aim was to investigate the CVD events (CVDEs) and survival between the patients with FMF-related AA amyloidosis and glomerulonephropathies (GN) to define possible predictors for CVDEs. A prospective follow-up study with FMF-amyloidosis and glomerulonephropathy (GN) was performed and patients were followed for CVDEs. Flow-mediated dilatation (FMD), FGF-23, serum lipid, hsCRP levels, BMI and HOMA were assessed. A Cox regression analysis was performed to evaluate the risk factors for CVDEs. There were 107 patients in the FMF-amyloidosis group and 126 patients with GN group. Forty-seven CVDEs were observed during the 4.2-years follow up; all 28 patients in the FMF-amyloidosis group and 14/19 patients with GN developed CVDEs before the age of 40 (p = 0.002). CVD mortality was 2.8 times higher (95% CI 1.02–7.76) in patients with FMF-amyloidosis. Across both groups, FMD and FGF23 (p < 0.001) levels were independently associated with the risk of CVDEs. Patients with FMF-amyloidosis are at increased risk of early CVDEs with premature mortality age. FGF 23, FMD and hsCRP can stratify the risk of early CVD in patients with FMF-related AA amyloidosis.

## Introduction

Familial Mediterranean fever (FMF) is the most common monogenic autoinflammatory disease in the world, with over 100,000 affected individuals. It is particularly common in individuals from the eastern Mediterranean Basin, where the disease has a prevalence of 100–200 per 100,000^1^. The gene for FMF, *MEFV,* was identified in 1997^2^. *MEFV* codes for the protein pyrin, which is a component of inflammasome function during inflammatory response and thus production of interleukin-1β (IL-1β). Prophylaxis with colchicine is the mainstay of treatment to prevent inflammatory attacks in FMF^3,4^. However, subclinical inflammation often continues during attack-free periods in patients with FMF. The most serious complication of sustained inflammation in FMF is AA amyloidosis, which leads to proteinuric chronic kidney disease (CKD)^5^. The typical clinical presentation of amyloidosis in FMF is kidney dysfunction, with progression from proteinuria to nephrotic syndrome to kidney failure and end-stage renal disease.

Renal AA amyloidosis with end-stage renal disease is strongly associated with excess mortality in individuals with FMF, especially due to cardiac complications^6–8^. Increased cardiovascular disease (CVD) risk in patients with FMF may also be related to chronic inflammation^7,9,10^. CVD is also the main cause of morbidity and mortality in patients with glomerulonephropathies (GN)^11^. Elevated inflammatory response and increased proteinuria are considered among the important causes of this increased risk^12^. Our previous studies also revealed that vascular inflammation and endothelial dysfunction are the main contributors for CVD events in this patient populations^7,13–15^.

There are a few published studies focused on CVD-related clinical outcomes in patients with FMF-related AA amyloidosis due to rarity of disease. We believe that our cohort is the largest collection of FMF patients with AA amyloidosis reported so far. We hypothesized that patients with glomerular disease, particularly those with FMF related AA amyloidosis, have higher prevalence of CVD event. Therefore, we sought to compare the prevalence of CVD-events and survival between the patients with proteinuria caused by FMF-related amyloidosis and GN. Secondly, we aimed to measure different markers of inflammation and endothelial dysfunction to define predictors of CVD events in our cohort.

## Material and method

### Patients

**Figure 1:**
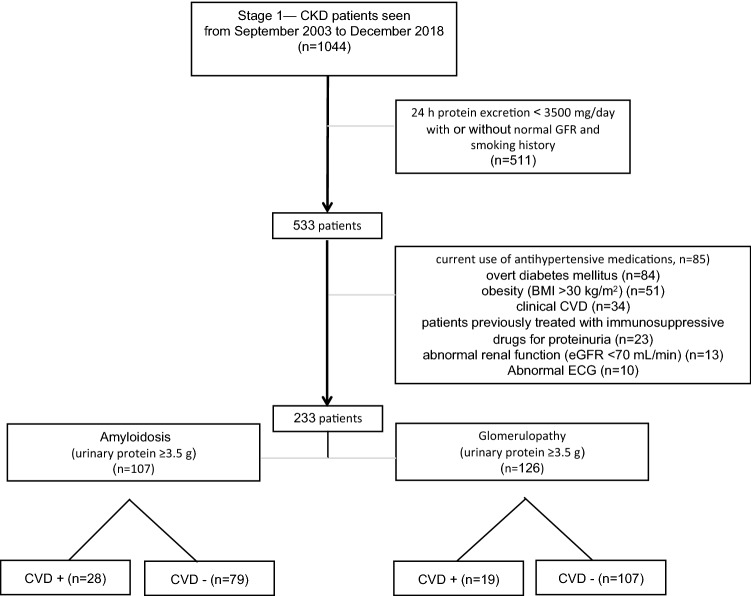
Flow diagram of patient selection for the study.

In this case cohort study, patients were recruited from the renal unit at Epigenetic Health Center Outpatient Clinics and Gulhane School of Medicine Ankara, Turkey between September 2003 and December 2018. The local ethical committee of Gulhane School of Medicine approved the study protocol and informed consent was obtained from each subject. All research was performed in accordance with approved guidelines/regulations. Patients who had AA amyloidosis and glomerulonephropathy were followed by a comprehensive patient-based registry, which was established at Gulhane School of Medicine in 2003 and is described in our previous studies with more details^16–18^. Among a referred population of 1044 patients, we excluded patients with less than 3500 mg proteinuria in 24 h, untreated hypertension (according to the JNC VII criteria^19^ and/or the current use of antihypertensive medications), overt diabetes mellitus, obesity (BMI > 30 kg/m^2^), smoking, clinical history of CVD (defined as the presence or history of ischemic heart disease, peripheral vascular disease and/or a cerebrovascular event), treatment with immunosuppressive drugs for their proteinuria, and abnormal renal function (eGFR < 70 mL/min) (Fig. 1).

In total, 233 patients were eligible for inclusion in the study. All patients had nephrotic-range proteinuria and normal GFR. A kidney biopsy was performed in all included patients (n = 233). FMF-related amyloidosis was diagnosed with the positive staining pattern with Congo red dye. According to the renal biopsy results and standard histological criteria^20^, all recruited patients were assigned to two possible groups: FMF-related amyloidosis (n = 107) and those with other GN including minimal change disease (MCD), focal segmental glomerulosclerosis (FSGS), membranous nephropathy (MN), membranoproliferative glomerulonephritis (MPGN), IgA nephropathy, secondary focal segmental glomerulosclerosis (sFSGS), lupus nephritis, and mesangial proliferation (MP) (n = 126), respectively. Patients were also compared according to their CVD status (positive and negative). Tell–Hashomer criteria were used for the diagnosis of FMF^21^. Patients with amyloidosis were already diagnosed in peripheral centers and were on colchicine treatment when they referred to our clinic.

### Clinical and cardiovascular assessment

All enrolled subjects were evaluated by standard physical examination, chest X-ray, baseline electrocardiogram, two-dimensional echocardiography, and routine biochemical laboratory tests, including liver and kidney function tests and 24-h urinary protein measurements. Flow mediated dilatation (FMD) and venous blood samples were taken following a 2-week wash-out period, during which time no vasoactive drugs (including colchicine) were given.

### Laboratory measurements

Blood sampling was done after an overnight fast. Fasting plasma glucose (FPG), total protein, serum albumin, total cholesterol, HDL cholesterol and triglycerides were determined by enzymatic colorimetry on an Olympus AU 600 auto analyzer using reagents from Olympus Diagnostics, GmbH (Hamburg, Germany). LDL cholesterol was calculated by Friedewald’s formula^22^. Twenty-four hours proteinuria was determined by a turbidimetric test using trichloroacetic acid (TCA). The serum basal insulin value was determined using a coated tube method (DPC Inc., USA). Renal function was estimated by the modified Modification of Diet in Renal Disease (MDRD) formula in ml/min and expresses per 1.73 m^2^ of body surface area^23^. Homeostasis model assessment (HOMA) was computed as: Homeostasis model assessment—insulin resistance (HOMA-IR) = FPG (mg/dL) × immunoreactive insulin (IRI) (µIU/mL)/405^24^.

Serum total calcium was measured by the cresolphtalein complex one method using Menagent Calcium 60 s kits (Menarini Diagnostics, Florence, Italy). Serum phosphorus was measured by the ammonia molybdate complex method using Menagent Phosphofix kits (Menarini Diagnostics)^17^.

To measure 25OHVD, we used high-performance liquid chromatography kits following manufacturer’s instructions (ImmuChrom GmbH, Heppenheim, Germany. Quantification of 25-OH vitamin D3 was made by high-performance liquid chromatography system with UV (264 nm) detector (Thermo Electron, San Jose, CA, USA)^17^.

High sensitivity C-reactive protein levels in serum (hsCRP) were determined by turbidimetric fixed rate method by an automated analyzer (Olympus AU-2700, Mishima, Japan). All blood samples in patients with FMF were taken from the 2 weeks apart from the prior attack.

Intact FGF-23 was measured using an enzyme-linked immunosorbent assay according to the manufacturer’s protocol (Kainos Laboratories International, Tokyo, Japan). This second-generation, two-site, monoclonal antibody enzyme-linked immunosorbent assay has previously been shown to recognize the biologically active, intact FGF-23. The Kainos Intact FGF-23 assay has a lower limit of detection of 3 pg/mL and intra-assay and interassay coefficients of variation of less than 5%. The calculated overall intra-assay coefficient of variation was 2.5%, and the calculated overall inter-assay coefficient of variation was 2.8%. We measured all samples in duplicate^17^.

### Vascular assessment imaging

Arterial pressure was measured by a physician three times after a 15-min resting period in the morning, and mean values were calculated for systolic and diastolic pressures for all patients. The method for the vascular assessment met the criteria established by the International Brachial Artery Reactivity Task Force^25^.

We determined endothelial dysfunction (ED) according to Celermajer et al.^26^. Measurements were made by a single observer using an ATL 5000 ultrasound system (Advanced Technology Laboratories Inc., Bothell, WA., USA) with a 12-MHz probe. The subjects remained at rest in the supine position for at least 15 min before examination. The subject’s arm was comfortably immobilized in the extended position to allow consistent recording of the brachial artery 2–4 cm above the antecubital fossa. Three adjacent measurements of end-diastolic brachial artery diameter were made from single 2-D frames. All ultrasound images were recorded for subsequent blinded analysis. A pneumatic tourniquet was then inflated to 200 mmHg with obliteration of the radial pulse. After 5 min, the cuff was deflated. Flow measurements were made 60 s post-deflation. The maximum flow mediated dilatation (FMD_max_) diameters were calculated as the average of three consecutive maximum diameter measurements. The percent change in average FMD_max_ diameter compared with baseline resting diameter was defined as flow-mediated diameter change (FMD). The intra observer coefficient of variation for FMD was 5.1%.

### Statistical analysis

Statistical analysis was performed using Statistical Package of Social Science (SPSS) for Windows, version 15.0 (SPSS Inc, Chicago, IL). Descriptive statistics were represented as frequencies and percentages for categorical variables and median (minimum–maximum) for continuous variables as appropriate. Distributions of variables were evaluated by one sample Kolmogorov Smirnov test. Mann–Whitney U test were used to compare continuous variables between groups. Categorical variables compared by chi-square tests.

Univariate survival analysis was performed to compare CVD-free period by the Kaplan–Meier method and the differences between survival curves were evaluated by using the log-rank test. The variables which had a statistically significant effect on Hazard Ratio were included in the Cox proportional hazard method as a multivariate analysis. The model was then reduced by using the backward elimination method and the best fitting models were reported.

### Ethics approval

GMMA (50687469-1491-70), Ankara, Turkey.

## Results

### Patients

There were 126 patients (77 male [61.1%], median age was 38 [23 to 60] years) with proteinuria due to GN and 107 patients (65 male [60.7%], median age was 36 [22 to 49] years) with proteinuria due to renal amyloidosis secondary to FMF.

Reported signs and symptoms of the patients with FMF-related amyloidosis who were under treatment with colchicine were fever (36.4%), abdominal pain (32.6%), myalgia (31.7%), arthritis (28.9%), pleuritis (18.7%), erysipelas (15.8%), and peritonitis (11.2%). Family history of FMF or amyloidosis was present in 29.9% and 22.4% of the patients, respectively. Appendectomy was performed in a quarter of patients (25.2%).

The primary etiologies of proteinuria according to renal biopsy in the non-amyloidosis patients were MCD (n = 31, 24.6%), FSGS (n = 28, 22%), MN (n = 19, 15.1%), MPGN (n = 15, 11.9%), IgA nephropathy (n = 11, 8.7%), sFSGS (n = 9, 7.1%), lupus nephritis (n = 7, 5.5%), and MP (n = 6, 4.7%).

### Clinical and laboratory characteristics

Comparisons of the clinical and biochemical characteristics between patients with proteinuria secondary to FMF-related amyloidosis and those with GN are presented in Table 1. Median age of diagnosis in patients with FMF-related amyloidosis was 15 (6 to 25) and 72.9% of patients were 18 or younger at the time of diagnosis of amyloidosis. Body mass index (BMI), systolic and diastolic blood pressure (BP), metabolic markers, and HOMA, were similar between the groups (p > 0.05). Triglyceride, P and PTH levels were higher in patients with FMF-related amyloidosis (p < 0.001, p < 0.001 and p = 0.004, respectively). Ca levels were higher in patients with GN (p < 0.001). There were no significant differences in proteinuria, GFR, FGF23 and 25OH-vitamin D levels among the groups. The hsCRP was significantly higher in FMF-related amyloidosis (p = 0.001). The FMD percentage and albumin levels were significantly lower in the FMF-related amyloidosis group (p < 0.001 and p = 0.001 respectively).

**Table 1:** Clinical and biochemical characteristic of patients with proteinuria secondary to FMF-related AA amyloidosis and with GN.

	Glomerulonephropathy (n = 126)		Amyloidosis (n = 107)		p
	Median	Min–max	Median	Min–max	
Age of diagnosis (years)	14.0	4–28	15.0	6–25	
**Age (years)**	38.0	23–60	36.0	22–49	0.010
Follow-up duration (months)	42.0	12–48	43.0	15–52	0.480
SBP (mm/hg)	134.0	113–148	134.0	112–140	0.318
DBP (mm/hg)	85.0	72–96	86.0	63–95	0.830
BMI (kg/m^2^)	27.0	21.2–30	27.0	19–32	0.664
**FMD (%)**	6.7	4–8.4	6.0	4–8	< 0.001
FGF23 (pg/dL)	40.1	18.6–88.6	40.6	21–99.6	0.718
**hsCRP (mg/L)**	18.0	8.5–32	19.0	9–63	0.001
Cholesterol (mg/dL)	273.0	162–448	284.0	184–372	0.071
**Triglyceride (mg/dL)**	192.0	82–284	219.0	25–369	< 0.001
LDL (mg/dL)	158.0	69–224	158.0	100–242	0.265
HDL (mg/dL)	48.0	31–58	48.0	32–58	0.580
Glucose (mg/dL)	81.0	50–112	88.0	50–109	0.052
Insulin (μUI/mL)	12.0	6.2–35	12.4	4.7–42	0.356
HOMA	2.4	1–7.3	2.5	1–10.4	0.123
**Ca (mg/dL)**	8.9	8.0–11.0	8.5	7.0–11.0	< 0.001
**P (mg/dL)**	4.0	2.0–6.0	4.9	2.0–6.0	< 0.001
**PTH (pg/dL)**	49.0	19.0–100.0	56.0	19.0–188.0	0.004
25OHVitD (nmol/dL)	51.2	30.2–90.6	50.3	19.7–87	0.207
**Albumin (g/dL)**	3.5	1.4–4.8	3.1	1.1–4.6	0.001
Proteinuria (g/24 h)	5275.0	3010–14,000	5600.0	3500–18,900	0.077
GFR (mL/min/1.73 m^2^)	87.5	75–125	90.0	75–105	0.060

*SBP* systolic blood pressure, *DBP* diastolic blood pressure, *BMI* Body Mass Index, *FMD* Flow-mediated dilatation, *hsCRP* high sensitivity C reactive protein, *LDL* low-density lipoprotein, *HDL* high-density lipoprotein, *HOMA* homeostasis model assessment, *Ca* calcium, *P* phosphate, *PTH* parathyroid hormone, *25OHVD* 25 hydroxy-vitamin D, *GFR* glomerular filtration rate.

### Follow-up for CVD events

Survival was determined from the day of examination, with a mean follow-up period of 42 (range 12 to 52) months. There was no loss to follow-up of any patient. The median follow-up time for patients with FMF-related amyloidosis was 43 (min.–max.: 15–52) months, and 42 (min.–max.: 12–48) months for patients with GN. CVD occurred in 47 patients; 28 patients with FMF-related amyloidosis and 19 patients with GN. Of these, 36 patients had one or more myocardial infarctions, 19 angina pectoris, 8 positive for coronoray artery stenosis which was verified by percutaneous transluminal coronary angiographie, and 9 left ventricular dysfunction. Seven patients had cerebrovascular disease and 4 had signs of peripheral atherothrombotic vascular disease. The distribution of CVDE among the GN group were MCD (n = 6), MPGN (n = 4), FSGS (n = 4), secondary FSGS (n = 2), MN (n = 1), Ig A nephropathy (n = 1), and in lupus nephritis (n = 1). All 28 patients who developed CVD events in the FMF-related amyloidosis versus 14/19 patients with GN developed CVD before 40 years of age (p = 0.002). Cardiovascular mortality was defined as death as a result of coronary heart disease (n = 10), sudden death (n = 4), stroke (n = 2), or complicated peripheral vascular disease (n = 1). CVD mortality was 2.8 times higher (95% CI 1.02–7.76, p = 0.03) in patients with FMF-related amyloidosis (n = 12) than GN (n = 5). Mortality due to CVD was higher in patients less than 40 years old with amyloidosis than GN (12/107 and 3/126 respectively, RR = 4.71, 95% CI 1.36–16.25, p = 0.006).

Table 2 shows the clinical and biochemical characteristics of patients in each group according to their CVDE status. FGF23 levels were significantly higher in patients with CVDEs in both groups compared to patients without CVDEs (p = 0.000 and p = 0.000 respectively). No significant difference in FGF23 level was found between CVD positive patients with FMF-related and GN (p = 0.551). FMD percentage was significantly lower in patients with CVDEs in both groups compared to patients without CVDEs (p = 0.000 and p = 0.000 respectively). FMD percentage was significantly lower, and CRP levels significantly higher in CVD positive patients with FMF-related amyloidosis than those with GN (p = 0.004 and p = 0.020, respectively).

**Table 2:** Clinical and biochemical characteristic of both study groups according to CVD status.

	Glomerulonephropathy		Amyloidosis		p*			
	CVD (−) (n = 107) a	CVD (+) (n = 19) b	CVD (−) (n = 79) c	CVD (+) (n = 28) d	a–b	c–d	a–c	b–d
Age (years)	37.0 (23.0–60.0)	38.0 (23.0–50.0)	36.0 (22.0–49.0)	37.0 (23.0–40.0)	0.478	0.881	0.035	0.063
Follow-up duration (months)	43.0 (12.0–48.0)	34.0 (16.0–46.0)	44.0 (15.0–52.0)	34.5 (15.0–51.0)	0.004	0.006	0.263	0.753
SBP (mm/hg)	134.0 (113.0–148.0)	133.0 (113.0–142.0)	134.0 (114.0–140.0)	134.0 (112.0–138.0)	0.301	0.645	0.296	0.827
DBP (mm/hg)	85.0 (72.0–96.0)	85.0 (78.0–93.0)	87.0 (78.0–95.0)	85.0 (63.0–95.0)	0.732	0.591	0.876	0.983
BMI (kg/m^2^)	27.0 (21.2–30.0)	26.6 (23.0–30.0)	26.0 (19.0–32.0)	27.0 (23.5–32.0)	0.428	0.222	0.333	0.321
FMD (%)	6.8 (5.0–8.4)	6.1 (4.0–8.0)	6.3 (4.7–8.0)	5.0 (4.0–7.0)	0.000	0.000	0.000	0.004
FGF23 (pg/dL)	38.3 (18.6–79.4)	65.6 (18.7–88.6)	37.0 (21.0–83.3)	65.7 (25.4–99.6)	0.000	0.000	0.427	0.551
hsCRP (mg/L)	18.0 (8.5–25.0)	18.0 (12.0–32.0)	18.0 (9.0–48.0)	22.0 (13.0–63.0)	0.159	0.000	0.044	0.020
Cholesterol (mg/dL)	274.0 (162.0–448.0)	264.0 (183.0–362.0)	284.0 (193.0–372.0)	273.5 (184.0–372.0)	0.300	0.067	0.033	0.625
Triglyceride (mg/dL)	192.0 (82.0–284.0)	190.0 (116.0–258.0)	227.0 (25.0–369.0)	216.5 (115.0–257.0)	0.910	0.369	0.001	0.104
LDL (mg/dL)	155.0 (69.0–224.0)	165.0 (94.0–222.0)	158.0 (100.0–242.0)	163.0 (122.0–242.0)	0.255	0.178	0.358	0.931
HDL (mg/dL)	48.0 (31.0–58.0)	46.0 (34.0–57.0)	48.0 (32.0–58.0)	48.0 (38.0–57.0)	0.193	0.459	0.292	0.234
Glucose (mg/dL)	81.0 (50.0–112.0)	75.0 (50.0–105.0)	88.0 (50.0–109.0)	88.0 (67.0–105.0)	0.375	0.356	0.258	0.031
Insulin (μUI/mL)	11.0 (6.2–35.0)	15.7 (8.2–24.0)	12.0 (4.7–38.8)	16.0 (5.0–42.0)	0.112	0.123	0.525	0.664
HOMA	2.3 (1.0–7.3)	2.4 (1.1–4.9)	2.5 (1.0–8.4)	3.5 (1.2–10.4)	0.491	0.071	0.369	0.197
Ca (mg/dL)	8.9 (8.0–11.0)	10.0 (8.0–11.0)	8.2 (7.0–9.0)	9.85 (8.0–11.0)	< 0.001	< 0.001	< 0.001	0.367
P (mg/dL)	4.0 (2.0–6.0)	6.0 (4.0–6.0)	4.7 (2.0–6.0)	5.8 (3.0–6.0)	< 0.001	< 0.001	< 0.001	0.834
PTH (pg/dL)	48.0 (19–100.0)	69.0 (21.0–90.0)	52.0 (19.0–114.0)	98 (33.0–188.0)	< 0.001	< 0.001	0.02	0.01
25OHVitD (nmol/dL)	51.2 (30.2–90.6)	52.0 (35.5–65.7)	50.9 (23.3–87.0)	44.5 (19.7–76.0)	0.576	0.079	0.588	0.197
Albumin (g/dL)	3.5 (2.0–4.8)	3.6 (1.4–4.3)	3.2 (1.6–4.6)	3.0 (1.1–4.6)	0.891	0.459	0.003	0.224
Proteinuria (g/24 h)	5020.0 (3010.0–12,300.0)	6300.0 (3400.0–14,000.0)	4705.0 (3500.0–15,450.0)	7850.0 (4275.0–18,900.0)	0.001	0.000	0.782	0.059

*SBP* systolic blood pressure, *DBP* diastolic blood pressure, *BMI* Body Mass Index, *FMD* Flow-mediated dilatation, *hsCRP* high sensitivity C reactive protein, *LDL* low-density lipoprotein, *HDL* high-density lipoprotein, *HOMA* homeostasis model assessment, *Ca* calcium, *P* phosphate, *PTH* parathyroid hormone, *25OHVD* 25 hydroxy-vitamin D, *GFR* glomerular filtration rate.

*p < 0.01 considered as statistically significant.

**Figure 2:**
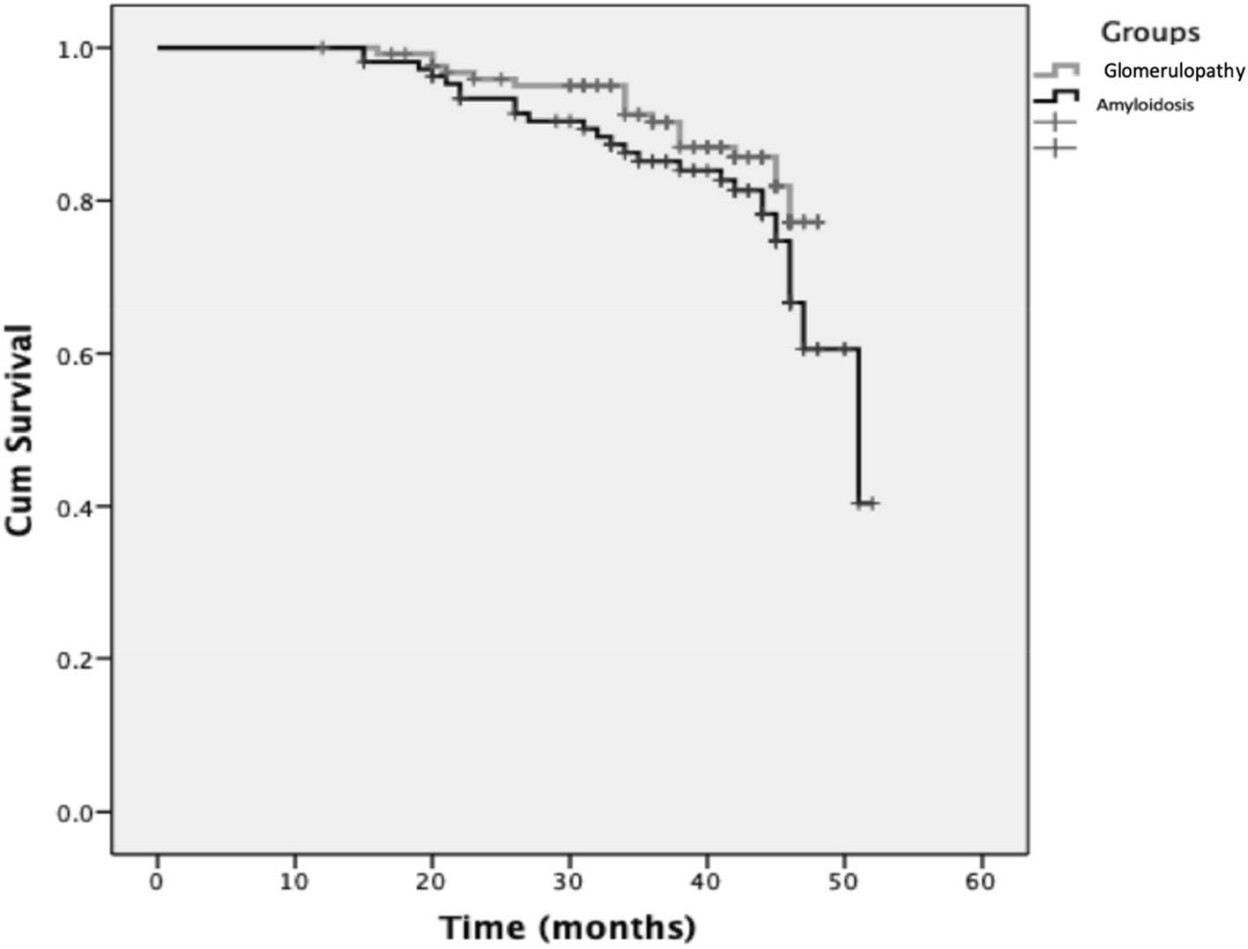
Comparison of cardiovascular disease survival between patients with FMF-related amyloidosis or glomerulonephropathy.

According to Kaplan–Meier survival analyses, patients with FMF-related amyloidosis were more likely to suffer a CVDE than patients with GN. The 3 years survival probability was 90% for the GN group and 83% for the amyloidosis group (Fig. 2). A Cox regression analysis was performed to evaluate the probability of CVDEs associated with each risk factors. Across both groups, FGF23 (HR 1.034 (95% CI 1.017–1.051)) and FMD (HR 0.388 (95% CI 0.262–0.575)) independently contributed to the risk of CVDEs. For patients with GN, FGF23 (HR 1.051 (95% CI 1.019–1.084)) and FMD (HR 0.522 (95% CI 0.300–0.908)) and for patients with amyloidosis, FGF23 (HR 1.035 (95% CI 1.012–1.058)), FMD (HR 0.216 (95% CI 0.109–0.430)) and hsCRP (HR 0.961 (95% CI 0.915–1.009)) independently contributed to the risk of CVDEs (Table 3).

**Table 3:** Multivariate analysis of factors associated with the risk of suffering a cardiovascular event.

	Variables	B	HR	95.0% CI for Exp (B)		p
				Lower	Upper	
All groups	FGF23	0.033	1.034	1.017	1.051	< 0.001
	FMD	− 0.946	0.388	0.262	0.575	< 0.001
Glomerulonephropathy	FGF23	0.050	1.051	1.019	1.084	0.002
	FMD	− 0.651	0.522	0.300	0.908	0.021
Amyloidosis	FGF23	0.034	1.035	1.012	1.058	0.003
	FMD	− 1.531	0.216	0.109	0.430	< 0.001
	hsCRP	− 0.040	0.961	0.915	1.009	0.108

*FMD* flow-mediated dilatation, *hsCRP* high sensitivity C reactive protein, *CI* Confidence interval.

## Discussion

Systemic inflammation in autoinflammatory disorders cause secondary systemic AA amyloidosis, which has been suggested as an important contributing factor to the excess CVD risk in patients with FMF^7^. Suppression of episodes of overt and subclinical inflammation with appropriate prophylactic treatment with colchicine or biological agents is recommended to reduce the risk of amyloidosis and cardiovascular risk in patients with FMF^1,27^. Therefore, it was hypothesised that patients with FMF-related amyloidosis could have higher risk of developing cardiovascular disease events. We found that patients with AA amyloidosis secondary to FMF have a significant risk of developing CVD events and early mortality. We also found that FMD and FGF 23 are the strongest predictors of CVD-event risk in patients with FMF amyloidosis. A timely diagnosis of FMF is important, as untreated disease has severe effects on quality of life and carries a risk of potentially fatal cardiovascular disease.

In our cohort, all patients with FMF-related amyloidosis who developed CVD events and cardiovascular-related death were less than 40 years old. Our long term longitudinal follow up study confirms that cardiovascular risk in this cohort is largely attributable to inflammation and diminished vascular function, and is the first to confirm prospectively that hsCRP, FGF23 and decreased FMD are independent predictors of mortality at an early age in patients with FMF. There are several studies demonstrating that CVD risk is higher in patients with rheumatoid arthritis, psoriatic arthritis and ankylosing spondylitis when compared to the general population^28–30^. There is no available longitudinal data in the literature about CVD risk in FMF-related amyloidosis or other autoinflammatory diseases. This study demonstrates that CVD related mortality rates are significantly higher in patients with FMF-related amyloidosis than those with GN, with relative risk 2.8. More specifically, CVD, CVDEs and premature mortality due to CVEs are increased in FMF-related amyloidosis as a consequence of a common process of genetic components, persistent inflammation and endothelial damage. Early identification of patients with FMF and FMF-related amyloidosis at high risk of CVD is essential.

Endothelial dysfunction and arterial stiffness are recognized as surrogate measurements of CVD. Increased common carotid intima-media thickness and impaired FMD in RA patients has reported in several studies to show endothelial dysfunction^31^. In our study, FMD was lowest in patients with FMF-related amyloidosis who developed CVD events. This is in keeping with work by Akdogan et al. showing that FMD was reduced, and carotid artery intima media thickness was increased, in patients with FMF compared to healthy controls^32^. Caliskan et al. showed that coronary microvascular function and left ventricular diastolic function were impaired in patients with FMF^33^. Decreased FMD has been associated with increased risk of CVDEs in many autoimmune rheumatological diseases^34–38^. Our study demonstrates that FMD measurement may be useful to assess CVD risk in patients with FMF-related amyloidosis; low FMD was a predictor of CVD mortality. Patients with decreased FMD should be considered as having high risk and should be treated more aggressively.

Proinflammatory cytokines have been implicated in endothelial dysfunction in FMF. We previously showed that endothelial function is more severely impaired in FMF patients with amyloidosis^18^. Elevated proinflammatory cytokine patterns during remissions indicate continuous subclinical inflammation that can induce endothelial injury between FMF flares^7^. In this study, we found that FGF-23 was markedly increased in patients with CVDEs. Recent data indicate that elevated circulating levels of FGF23 are independently associated multiple adverse cardiovascular outcomes in patients with chronic kidney disease (CKD) including vascular dysfunction^16^, left ventricular hypertrophy^39^, subclinical atherosclerosis, and mortality^39–41^.

The control of subclinical inflammation is a major goal in FMF treatment. In clinical practice, CRP is one of the most commonly used acute phase reactants to monitor FMF-activity, and is significantly increased during attacks^4^. In our cohort, we found that hsCRP levels were higher in patients with FMF-amyloidosis than those with GN, and CVD positive patients with FMF-amyloidosis had the highest hsCRP levels. Patients with FMF who developed amyloidosis are often resistant or non-compliant to treatment with colchicine and have high acute inflammatory markers between attacks. Increased serum CRP is correlated with impaired LV diastolic function and lower coronary flow reserve in patients with FMF^33^. It is evident from the present study that hsCRP is one of the most important biomarkers to predict CVDEs in FMF patients with amyloidosis. Every effort should be made to ensure early diagnosis, treatment (with colchicine or biologics), tight control of inflammation and close monitoring in order to prevent CVD in this population.

Serum AA amyloidosis is rare types of amyloidosis involve the heart. Myocardial involvement is the main predictor of outcome with the high mortality rate in patients with systemic amyloidosis^42,43^. In suspected patients for cardiac amyloidosis, transthoracic echocardiography is a cornerstone and essential for the initial evaluation^44^. Cardiac magnetic resonance imaging is not routine evaluation technique and assists in establishing heart involvement^45^. It has great potential for prognosis in patients with systemic amyloidosis and provides accurate anatomical and functional assessment of the myocardium. We did not screen our patients with cardiac imaging techniques, and cardiac amyloidosis may be a main contributing factor for the mortality. Implementation of cardiac imaging in patients with FMF related AA amyloidosis can improve our knowledge of cardiac amyloidosis and lead to early diagnosis and more decent patient outcome.

Our study has several strengths and limitations. Our cohort is the largest FMF related amyloidosis cohort to date. Possible confounding factors including hypertension, diabetes mellitus, obesity, medication and renal failure were excluded from the study but we are aware that there are still many confounders such as cytokine levels. Well-monitored follow-up data enabled us to analyze patients with similar baseline risk for cardiovascular disease events, using a prospective case–control design. We were not able to perform ECHO to rule out cardiac amyloidosis in our cohort. Another limitation of our study to be mentioned that a possible lead-time bias due to disease duration.

In conclusion, there are few studies focusing on FMF-related amyloidosis and cardiovascular disease event risk. Due to the rarity of many autoinflammatory syndromes, little is known about their associated cardiovascular manifestations. Despite the lack of outcome data, our results clearly support that screening for CVD with FMD, FGF23, and hsCRP in patients with FMF and specifically in amyloidosis appears reasonable. Our findings highlight the importance of the higher than average mortality rate among patients with FMF-related amyloidosis. Ideally, effective control of subclinical inflammation will mitigate the risk of CVDEs for this group. Therefore, overall CVD risk assessment must be the ultimate goal to reduce the risk of premature mortality due to CVDEs in patients with FMF-related amyloidosis. Unfortunately, there are still many unmet needs in this field, as is reflected in above, but it is essential to take into account that early diagnosis and management of patients with FMF and AA amyloidosis and follow up CVD risk assessment must be key parts of routine clinical practice.

## References

[1] Ozen S, Demirkaya E, Erer B (2016). EULAR recommendations for the management of familial Mediterranean fever. Ann. Rheum. Dis..

[2] Consortium IF (1997). Ancient missense mutations in a new member of the RoRet gene family are likely to cause familial Mediterranean fever. Cell.

[3] Demirkaya E, Erer B, Ozen S (2016). Efficacy and safety of treatments in familial Mediterranean fever: A systematic review. Rheumatol. Int..

[4] Erer B, Demirkaya E, Ozen S (2016). What is the best acute phase reactant for familial Mediterranean fever follow-up and its role in the prediction of complications? A systematic review. Rheumatol. Int..

[5] Hull KM, Kastner DL, Balow JE (2002). Hereditary periodic fever. New Engl. J. Med..

[6] Pinney JH, Hawkins PN (2012). Amyloidosis. Ann. Clin. Biochem..

[7] Yilmaz MI, Demirkaya E, Acikel C (2014). Endothelial function in patients with familial Mediterranean fever-related amyloidosis and association with cardiovascular events. Rheumatology (Oxford, England).

[8] Twig G, Livneh A, Vivante A (2014). Mortality risk factors associated with familial Mediterranean fever among a cohort of 1.25 million adolescents. Ann. Rheum. Dis..

[9] Obici L, Merlini G (2012). Amyloidosis in autoinflammatory syndromes. Autoimmunol. Rev..

[10] Erken E, Erken E (2018). Cardiac disease in familial Mediterranean fever. Rheumatol. Int..

[11] Foley RN, Parfrey PS, Sarnak MJ (1998). Epidemiology of cardiovascular disease in chronic renal disease. J. Am. Soc. Nephrol..

[12] Agrawal V, Marinescu V, Agarwal M (2009). Cardiovascular implications of proteinuria: An indicator of chronic kidney disease. Nat. Rev. Cardiol..

[13] Kanbay M, Afsar B, Siriopol D (2016). Endostatin in chronic kidney disease: Associations with inflammation, vascular abnormalities, cardiovascular events and survival. Eur. J. Intern. Med..

[14] Gungor O, Unal HU, Guclu A (2017). IL-33 and ST2 levels in chronic kidney disease: Associations with inflammation, vascular abnormalities, cardiovascular events, and survival. PLoS ONE.

[15] Yilmaz MI, Romano M, Basarali MK (2020). The effect of corrected inflammation, oxidative stress and endothelial dysfunction on Fmd levels in patients with selected chronic diseases: A quasi-experimental study. Sci. Rep..

[16] Yilmaz MI, Sonmez A, Saglam M (2008). ADMA levels correlate with proteinuria, secondary amyloidosis, and endothelial dysfunction. J. Am. Soc. Nephrol..

[17] Yilmaz MI, Sonmez A, Saglam M (2010). FGF-23 and vascular dysfunction in patients with stage 3 and 4 chronic kidney disease. Kidney Int..

[18] Yilmaz MI, Demirkaya E, Acikel C (2014). Endothelial function in patients with familial Mediterranean fever-related amyloidosis and association with cardiovascular events. Rheumatology (Oxford, England).

[19] Chobanian AV, Bakris GL, Black HR (2003). The Seventh Report of the Joint National Committee on prevention, detection, evaluation, and treatment of high blood pressure: The JNC 7 report. JAMA.

[20] Westermark P, Benson MD, Buxbaum JN (2005). Amyloid: Toward terminology clarification. Report from the Nomenclature Committee of the International Society of Amyloidosis. Amyloid Int. J. Exp. Clin. Investig..

[21] Demirkaya E, Saglam C, Turker T (2016). Performance of different diagnostic criteria for familial Mediterranean fever in children with periodic fevers: Results from a multicenter international registry. J. Rheumatol..

[22] Friedewald WT, Levy RI, Fredrickson DS (1972). Estimation of the concentration of low-density lipoprotein cholesterol in plasma, without use of the preparative ultracentrifuge. Clin. Chem..

[23] Levey AS, Bosch JP, Lewis JB (1999). A more accurate method to estimate glomerular filtration rate from serum creatinine: A new prediction equation. Modification of diet in renal disease study group. Ann. Intern. Med..

[24] Matthews DR, Hosker JP, Rudenski AS (1985). Homeostasis model assessment: Insulin resistance and beta-cell function from fasting plasma glucose and insulin concentrations in man. Diabetologia.

[25] Corretti MC, Anderson TJ, Benjamin EJ (2002). Guidelines for the ultrasound assessment of endothelial-dependent flow-mediated vasodilation of the brachial artery: A report of the International brachial artery reactivity task force. J. Am. Coll. Cardiol..

[26] Celermajer DS, Sorensen KE, Gooch VM (1992). Non-invasive detection of endothelial dysfunction in children and adults at risk of atherosclerosis. Lancet (London, England).

[27] Akar S, Yuksel F, Tunca M (2012). Familial Mediterranean fever: Risk factors, causes of death, and prognosis in the colchicine era. Medicine.

[28] Agca R, Heslinga SC, van Halm VP (2016). Atherosclerotic cardiovascular disease in patients with chronic inflammatory joint disorders. Heart.

[29] Avina-Zubieta JA, Thomas J, Sadatsafavi M (2012). Risk of incident cardiovascular events in patients with rheumatoid arthritis: A meta-analysis of observational studies. Ann. Rheum. Dis..

[30] Jamnitski A, Visman IM, Peters MJ (2011). Prevalence of cardiovascular diseases in psoriatic arthritis resembles that of rheumatoid arthritis. Ann. Rheum. Dis..

[31] Szekanecz Z, Kerekes G, Der H (2007). Accelerated atherosclerosis in rheumatoid arthritis. Ann. N. Y. Acad. Sci..

[32] Akdogan A, Calguneri M, Yavuz B (2006). Are familial Mediterranean fever (FMF) patients at increased risk for atherosclerosis? Impaired endothelial function and increased intima media thickness are found in FMF. J. Am. Coll. Cardiol..

[33] Caliskan M, Gullu H, Yilmaz S (2007). Impaired coronary microvascular function in familial Mediterranean fever. Atherosclerosis.

[34] Chatterjee Adhikari M, Guin A, Chakraborty S (2012). Subclinical atherosclerosis and endothelial dysfunction in patients with early rheumatoid arthritis as evidenced by measurement of carotid intima-media thickness and flow-mediated vasodilatation: an observational study. Semin. Arthritis Rheum..

[35] Garg N, Krishan P, Syngle A (2016). Atherosclerosis in psoriatic arthritis: A multiparametric analysis using imaging technique and laboratory markers of inflammation and vascular function. Int. J. Angiol..

[36] Cugno M, Borghi MO, Lonati LM (2010). Patients with antiphospholipid syndrome display endothelial perturbation. J. Autoimmunol..

[37] Sincer I, Kurtoglu E, Yilmaz Coskun F (2015). Association between serum total antioxidant status and flow-mediated dilation in patients with systemic lupus erythematosus: An observational study. Anatol. J. Cardiol..

[38] Silva I, Teixeira A, Oliveira J (2015). Endothelial dysfunction and nailfold videocapillaroscopy pattern as predictors of digital ulcers in systemic sclerosis: A cohort study and review of the literature. Clin. Rev. Allergy Immunol..

[39] Gutierrez OM, Januzzi JL, Isakova T (2009). Fibroblast growth factor 23 and left ventricular hypertrophy in chronic kidney disease. Circulation.

[40] Gutierrez OM, Mannstadt M, Isakova T (2008). Fibroblast growth factor 23 and mortality among patients undergoing hemodialysis. N. Engl. J. Med..

[41] Faul C, Amaral AP, Oskouei B (2011). FGF23 induces left ventricular hypertrophy. J. Clin. Investig..

[42] Falk RH, Alexander KM, Liao R (2016). AL (light-chain) cardiac amyloidosis: A review of diagnosis and therapy. J. Am. Coll. Cardiol..

[43] Grogan M, Scott CG, Kyle RA (2016). Natural History of wild-type transthyretin cardiac amyloidosis and risk stratification using a novel staging system. J. Am. Coll. Cardiol..

[44] Papathanasiou M, Carpinteiro A, Rischpler C (2020). Diagnosing cardiac amyloidosis in every-day practice: A practical guide for the cardiologist. Int. J. Cardiol. Heart Vasc..

[45] Martinez-Naharro A, Treibel TA, Abdel-Gadir A (2017). Magnetic resonance in transthyretin cardiac amyloidosis. J. Am. Coll. Cardiol..

